# Age-related macular degeneration: suitability of optogenetic therapy for geographic atrophy

**DOI:** 10.3389/fnins.2024.1415575

**Published:** 2024-07-01

**Authors:** Grace A. Borchert, Hoda Shamsnajafabadi, Benjamin W. J. Ng, Kanmin Xue, Samantha R. De Silva, Susan M. Downes, Robert E. MacLaren, Jasmina Cehajic-Kapetanovic

**Affiliations:** ^1^Nuffield Laboratory of Ophthalmology, Department of Clinical Neurosciences, University of Oxford, Oxford, United Kingdom; ^2^Oxford Eye Hospital, Oxford University Hospitals NHS Foundation Trust, Oxford, United Kingdom

**Keywords:** optogenetic therapy, gene therapy, AMD, age-related macular degeneration, optogenetics

## Abstract

Age-related macular degeneration (AMD) is a growing public health concern given the aging population and it is the leading cause of blindness in developed countries, affecting individuals over the age of 55 years. AMD affects the retinal pigment epithelium (RPE) and Bruch’s membrane in the macula, leading to secondary photoreceptor degeneration and eventual loss of central vision. Late AMD is divided into two forms: neovascular AMD and geographic atrophy (GA). GA accounts for around 60% of late AMD and has been the most challenging subtype to treat. Recent advances include approval of new intravitreally administered therapeutics, pegcetacoplan (Syfovre) and avacincaptad pegol (Iveric Bio), which target complement factors C3 and C5, respectively, which slow down the rate of enlargement of the area of atrophy. However, there is currently no treatment to reverse the central vision loss associated with GA. Optogenetics may provide a strategy for rescuing visual function in GA by imparting light-sensitivity to the surviving inner retina (i.e., retinal ganglion cells or bipolar cells). It takes advantage of residual inner retinal architecture to transmit visual stimuli along the visual pathway, while a wide range of photosensitive proteins are available for consideration. Herein, we review the anatomical changes in GA, discuss the suitability of optogenetic therapeutic sensors in different target cells in pre-clinical models, and consider the advantages and disadvantages of different routes of administration of therapeutic vectors.

## Introduction

Age-related macular degeneration (AMD) is the leading cause of vision impairment over the age of 55 years in high-income countries (Collaborators GBaVI, 2021). With an aging population, there is an increasing prevalence of AMD and global burden of disease. AMD was estimated to affect 196 million people in 2020 and this is projected to increase to 288 million in 2040 (Wong et al., 2014). AMD affects the macula, which is responsible for central vision, and affects reading, recognizing faces and driving. There are significant psychosocial consequences associated with AMD with decreased independence and mobility, as well as reduced quality of life and increased risk of falls (Scott et al., 2016; Taylor et al., 2016).

Late AMD can be divided into neovascular AMD and geographic atrophy (GA). GA has a significant humanistic and economic burden (Sarda et al., 2021), the prevalence of GA in advanced AMD was 60% (Abdin et al., 2023), and average annual incidence of GA was 1.9 per 1,000 aged >50 years (Rudnicka et al., 2015). There are limitations in the management of GA secondary to AMD. Only two FDA approved treatments slow progression of GA: complement factor 3 inhibitor, pegcetacoplan, and complement factor 5 inhibitor, avacincaptad pegol (Heier et al., 2023; Khanani et al., 2023). Meanwhile, gene replacement therapy approaches, typically developed for monogenic disorders, are limited by the polygenic nature of AMD and genetic capacity of 4.7 kb for AAV-based vectors. These limitations highlight the need for an alternative approach to restore visual perception in GA.

Optogenetic therapy is a promising strategy to restore vision and to date it has been in development for patients affected by late-stage inherited retinal degenerations, such as retinitis pigmentosa. These patients typically have extensive pan-retinal degeneration and barely light perception vision. Optogenetic therapy takes advantage of their relatively unaffected inner retina and optic nerve, to introduce light-sensitive proteins to the remaining inner retinal cells and restore visual function (McClements et al., 2020). Following proof-of concept in preclinical models, several optogenetic therapies have progressed to clinical trials for end-stage retinal degeneration (Sahel et al., 2021).

Optogenetic approaches are agnostic to genetic causes of retinal degeneration which is relevant when there are multiple mechanisms underlying the pathogenesis of AMD (John et al., 2023). In this review, we discuss the feasibility of optogenetic therapy as a therapeutic strategy for GA secondary to AMD. We begin by discussing the retinal degeneration progression, suitable optogenetic sensors, target cells, models and routes of administration.

## Geographic atrophy changes secondary to AMD

**Figure 1:**
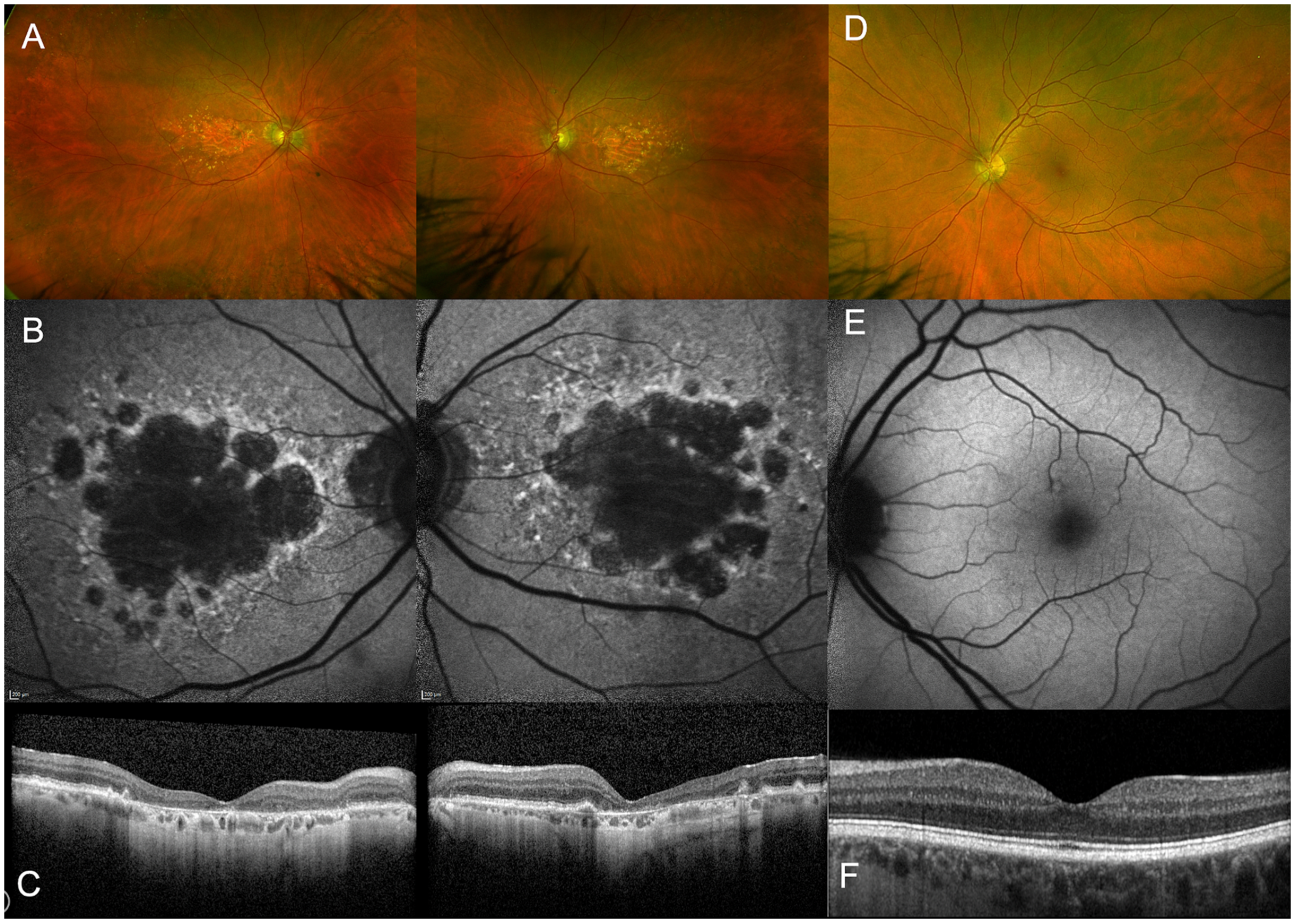
Multimodal imaging of a 90-year old patient with AMD-related geographic atrophy **(A–C)** compared to a healthy control **(D–F)**. Widefield retinal imaging by Optos shows atrophic patches located at the macula **(A)** which are not seen in healthy subjects **(D)**. Fundus autofluoresence imaging shows loss of RPE as demonstrated by dark areas **(B)**, not seen in a healthy control. Optical coherence tomography (OCT) shows a cross-section through macula demonstrating thinning of outer retinal layers **(C)** compared to normal retinal thickness in a healthy control **(F)**.

Geographic atrophy in AMD describes a well demarcated area of atrophy (Figure 1). On optical coherence tomography (OCT), GA usually corresponds to cRORA (complete RPE and outer retinal atrophy). This is defined as a zone of RPE disruption with hypertransmission at least 250 μm in diameter associated with loss of overlying photoreceptor (Sadda et al., 2018). Histological studies of GA described that the external limiting membrane delineates the area of the atrophic lesion (Li et al., 2018). The atrophic area itself has loss of photoreceptors, while the Henle fiber layer and outer nuclear layer have both halved in thickness in GA secondary to AMD (Li et al., 2018). However, there were no obvious changes noted in the inner retina. Around the edges of atrophic area, abundant macrophages and microglia have been observed, as well as depositions of complement factor H in Bruch’s membrane and membrane attack complex on RPE cells (Bonilha et al., 2020). GA has also been associated with choriocapillaris vascular loss in the center (McLeod et al., 2009), and a concentric ring of vascular loss (Choi et al., 2015). More specific retinal vascular changes have been reported with decreased vessel density in the superficial vascular complex, and intermediate and deep capillary plexus (You et al., 2020; Taylor et al., 2024).

**Figure 2:**
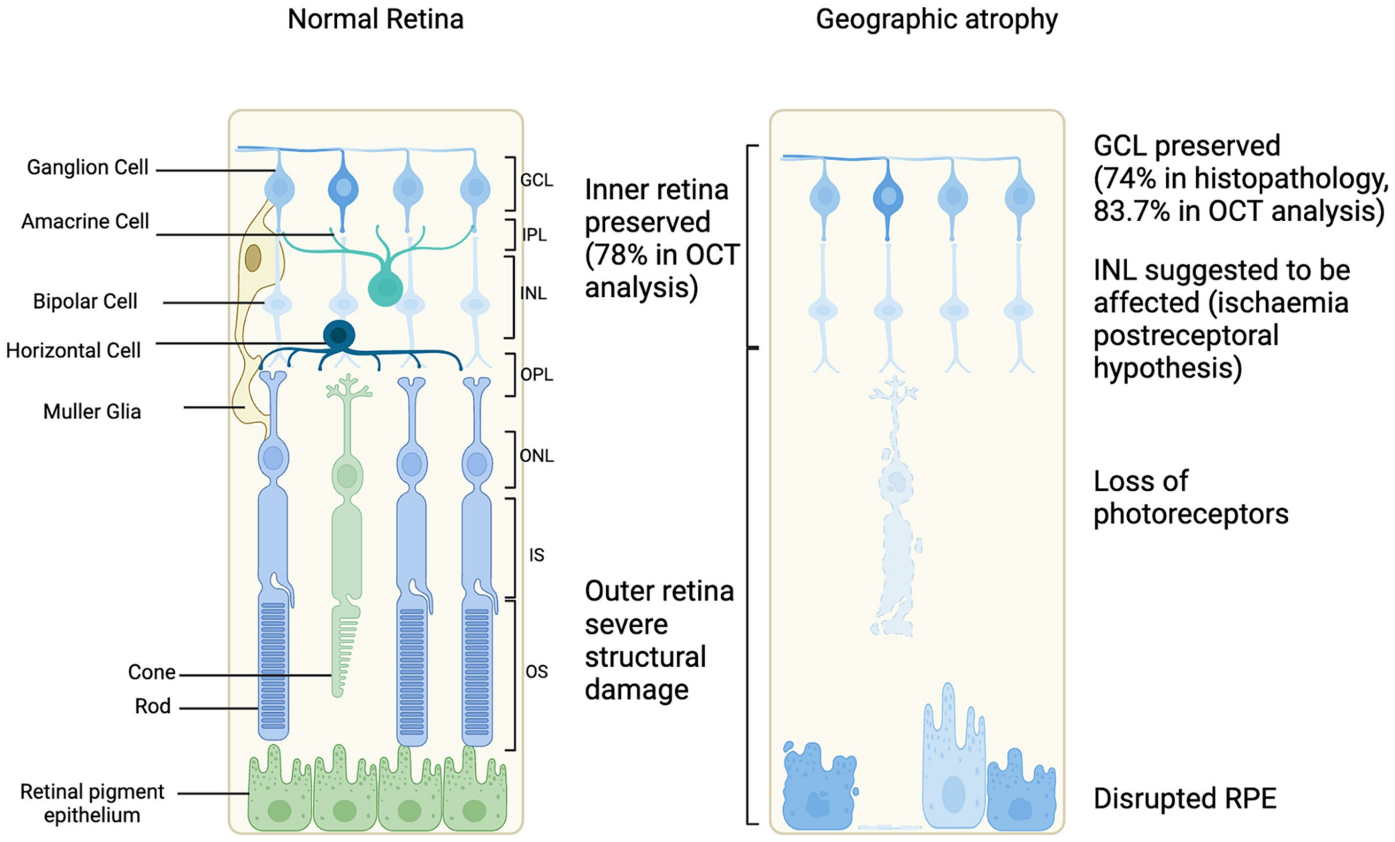
Schematic of cellular changes in geographic atrophy secondary to age-related macular degeneration progression compared to normal retina (Kim et al., 2002; Wolf-Schnurrbusch et al., 2008; Ramkumar et al., 2018).

From a cross-sectional view, OCT has had an important role in characterizing the progression of retinal degeneration as it depicts the affected layers. It has been reported that the integrity of inner retina is generally preserved in most GA affected eyes (40/52, 78%). Meanwhile, the outer retina had severe structural changes based on OCT as illustrated in Figure 2 (Wolf-Schnurrbusch et al., 2008). Discrete hyperreflective loci on SD-OCT has been suggested to represent RPE migration in the outer retinal layers to ectopic areas in the inner retina, have been considered a good biomarker for progression to GA (Christenbury et al., 2013). In GA, OCT findings show a disrupted RPE, hypertransmission and loss of photoreceptors. This collection of features has been defined as atrophy by the Classification of Atrophy Meeting (CAM) (Sadda et al., 2018). The hypertransmission effect is a result of increased signal penetration into the choroid following loss of RPE, outer retinal layers and choricocapillaris.

## Optogenetic target cell in geographic atrophy

**Figure 3:**
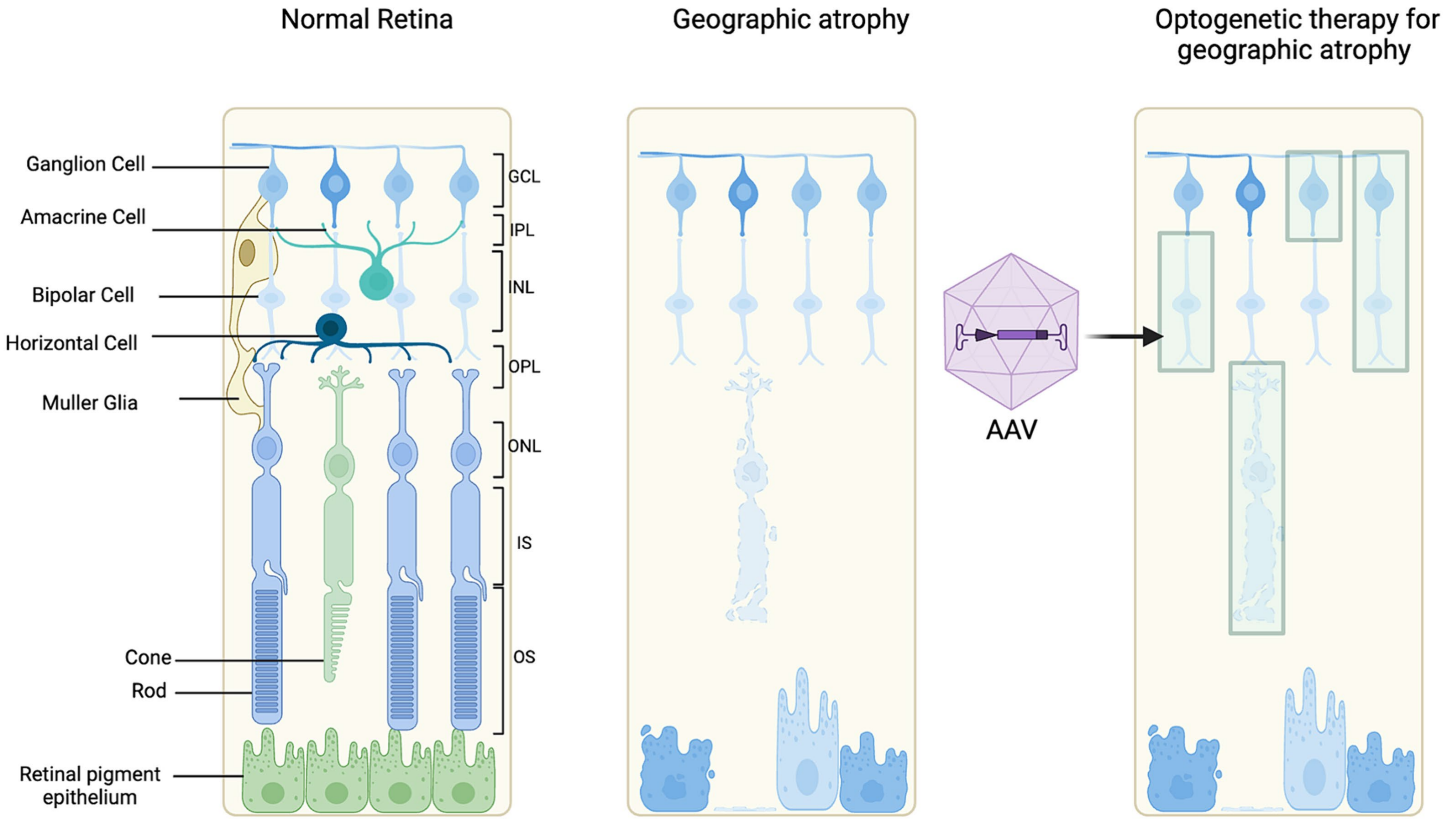
Schematic of optogenetic target cells. Illustrating a normal retina for reference, retinal degeneration and optogenetic therapy treated retinal degeneration.

An understanding of the retinal layers affected in GA secondary to AMD is important to guide investigation of an appropriate target cell for optogenetic therapy (Figure 3) (McClements et al., 2020). The feasibility, advantages and disadvantages of target cells will be discussed further (Table 1).

**Table 1:** Comparison of bipolar and ganglion cell as a target for optogenetic therapy.

	Bipolar cell	Ganglion cell
Geographic atrophy in AMD	Can become affected depending on severity and extent of GA	Typically, remains intact based on histopathology and OCT analysis
Generic advantages as an optogenetic target in retinal degeneration	Early, spatial and temporal processing Potential restoration of intrinsic visual processing (ON and OFF pathways) Signal amplification	More efficient optogene expression by *in vitro* electrophysiological recordings and animal behavior (Lu et al., 2018) Survive in advanced GA Direct connection to the brain and complex visual processing Easy access compared to bipolar cells
Generic disadvantages as an optogenetic target in retinal degeneration	More susceptible to damage Indirect stimulation of ganglion cells for visual processing Difficulty achieving optimal spatial resolution	Elements of visual processing not restored (ON/OFF pathways or center-surround organization) Less spatial resolution compared to bipolar cells
Visual response properties reported *in vivo*	More diversity of response types so could have more complex vision (Rodgers et al., 2023)	Improved visual response based on amplitude and response latency compared to bipolar cells (Rodgers et al., 2023) Reduced response diversity therefore simplified vision (Rodgers et al., 2023)
What we have learnt from retinal prostheses in geographic atrophy	Subretinal prostheses target bipolar cells (e.g., PRIMA bionic vision System) in GA Surgically challenging and time-consuming with risk of complications Implant was well-tolerated Provided visual acuity improvement up to 0.9 LogMAR in GA (Pixium Vision, 2021)	Epiretinal prostheses target ganglion cells (e.g., Argus II Retinal Prosthesis System, Second Sight Inc., United States for GA) Less complex surgery Argus II had FDA and CE mark approval so deemed safe (Ramirez et al., 2023) Central visual function was elicited by Argus II over GA area in all patients before and after resolution of adverse events (Stanga et al., 2017) Provided maximum of visual acuity was 20/1262 (6/379) (Cehajic Kapetanovic et al., 2020)

Bipolar cells are interneurons that transmit information from the photoreceptors to the ganglion cells. Following loss of the photoceptors, targeting the bipolar cells with an optogenetic tool could provide an alternative route to stimulate processing of visual signals (Cehajic Kapetanovic et al., 2014; Cehajic-Kapetanovic et al., 2014). Bipolar cells in the INL have been suggested to be affected in GA secondary to AMD due to the post-receptoral functional loss distal to photoreceptors (Feigl et al., 2007).

However, the advantages of targeting bipolar cells include early signal transduction, and improved spatial and temporal processing compared to ganglion cells. An AAV capsid mutation improved transduction of bipolar cells in *rd1* mouse and human retina *ex vivo* (De Silva et al., 2016). In optogenetic therapy for retinal degeneration, in the *rd1* mouse model, delivery of ChR2 with electroporation to the ON bipolar cells induced light-evoked activity in the ganglion cells and cortex (Lagali et al., 2008). A specific and stable expression of ChR2 was achieved in ON bipolar cells by injecting AAV8-Y733F virus packaged with an *mGRM6-SV40-hChR2-heGFP* vector in *rd10* mice. This resulted in electrophysiological responses from ganglion cells and visually guided behavior at 10 weeks and 10 months after injection (Doroudchi et al., 2011). Meanwhile, ChR delivered via AAV to ON bipolar cells restored ON and OFF responses at both the retinal and cortical level in blind mice (Cronin et al., 2014; Macé et al., 2015). The level of transgene expression in bipolar cells was the limiting factor since the electroporation resulted in low, transient gene expression.

In advanced stages of retinal degeneration in AMD, the innermost layer of the retina, the ganglion cells are preserved. Histopathology studies have reported 74.3% preservation of the ganglion cell layer (Kim et al., 2002). Further, the ganglion cell layer has also been assessed in patients with GA using OCT imaging, segmenting the layers and measuring the ganglion cell layer volume (Ramkumar et al., 2018). It was demonstrated that 83.7% of GA patients had a preserved ganglion cell layer (Ramkumar et al., 2018). This suggests that the therapies targeting ganglion cells may be feasible for restoring vision. The complex electrical signaling processes, such as center-surround organization or ON/OFF pathways, before the retinal ganglion cells would be bypassed which could be an advantage if affected in GA (Ichinose and Habib, 2022). Improving AAV delivery of optogene expression targeting retinal ganglion cells could be enhanced by a higher titer of vector, using regulatory elements or modifying the capsid to enhance transduction efficiency (Nieuwenhuis et al., 2023).

## Optogenetic sensor

**Figure 4:**
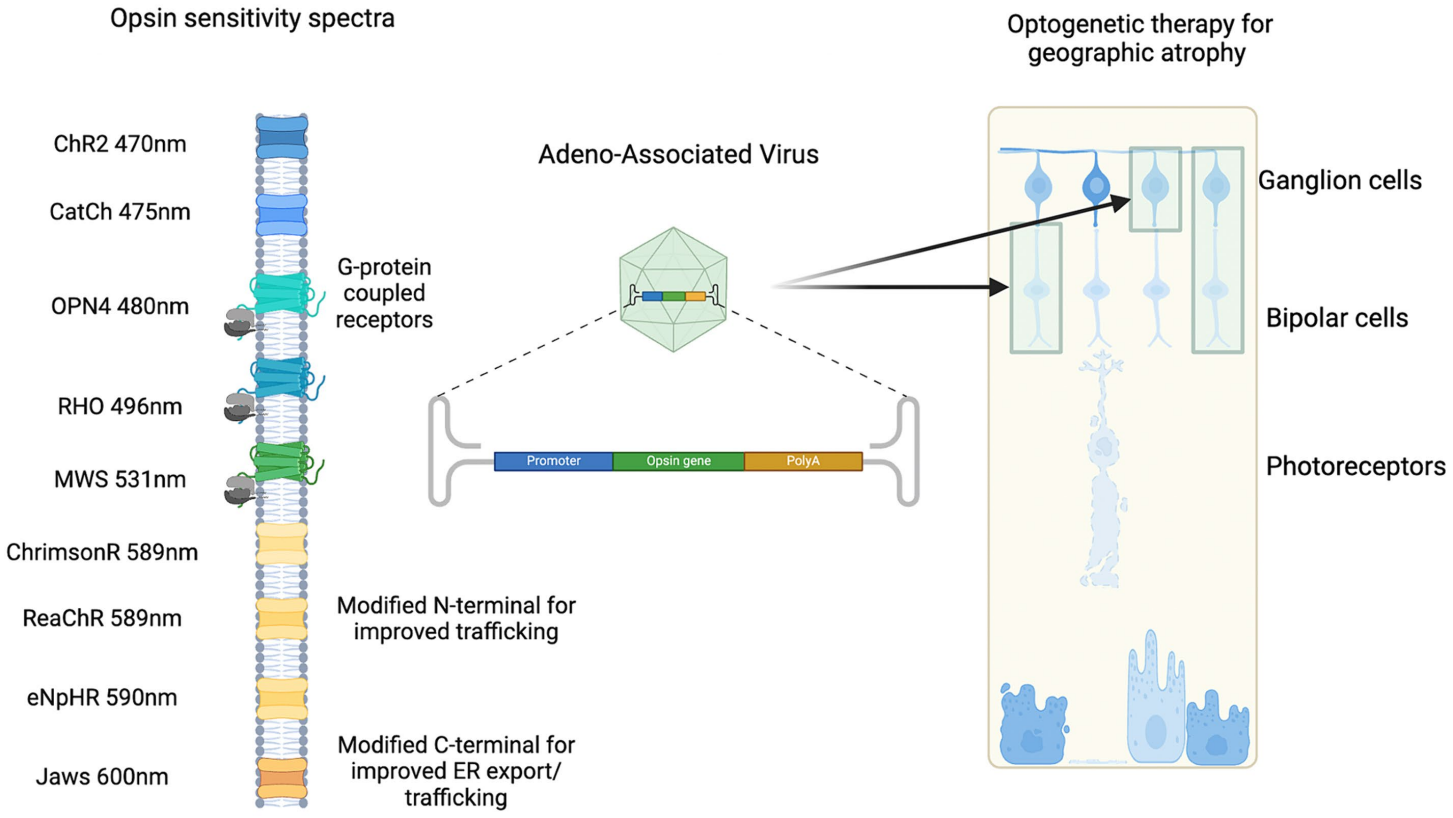
Examples of spectral sensitivity of optogenetic sensors delivered by gene therapy to target cells.

There are several factors that need to be considered in deciding a suitable optogenetic sensor in GA (Figure 4). These include light sensitivity, spectral sensitivity, and spatial resolution (Simon et al., 2020). Native human opsins have the advantage of eliciting less of an immune reaction. There are five main types that include rhodopsin (expressed by rod photoreceptors), short-wave cone opsin, medium-wave cone opsin, long-wave cone opsin (expressed by cone photoreceptors), and melanopsin (expressed in photosensitive retinal ganglion cells). Engineered structure-guided mutagenesis has enabled opsins to have high potency and faster kinetics (Sridharan et al., 2022). For example, ChroME2.0 opsins have demonstrated large-scale temporal and spatial control of neuronal activity *in vivo* which expands the patterns of illumination (Sridharan et al., 2022). Alternatively, luminopsins which are a luciferase fused to an opsin provide bimodal control of neurons with a chemogenetic and optogenetic approach (Slaviero et al., 2023).

Several optogenetic tools, including microbial and human opsin-based molecules, are being developed for vision restoration in advanced retinal degeneration (Gaub et al., 2018). Previous work has showed that AAV delivered ectopic human rhodopsin could restore visual function in advanced retinal degeneration of a *rd1* mouse model (Cehajic-Kapetanovic et al., 2015). Rhodopsin could be a relevant optogenetic sensor in AMD patients since there is delayed dark adaptation (Hogg and Chakravarthy, 2006).

Cone opsins or modified channel opsins, targeted to inner retinal neurons, could be useful to restore cone-like photoreception that has been lost in GA secondary to AMD. Further insights into optogenetic sensors have been investigated *in vivo*. One of the initial optogenetic studies used exogenous mouse melanopsin expression in remaining retinal cells and resulted in improved visual function in *rd1* mice (Lin et al., 2008). Since then, human melanopsin expression in the inner retina of *rd1* mice was evaluated (De Silva et al., 2017). There was greater light sensitivity and visual responses in *rd1* mice treated with human melanopsin versus controls with treatment effects persisting to over 12 months following injection (De Silva et al., 2017). Further, a chimera of mouse melanopsin and the mGluR6 receptor were tested in a transgenic mouse model delivered by AAV2 to target ON-bipolar cells, and showed a transduction of around 12% in bipolar cells and light responses recorded in ganglion cells (van Wyk et al., 2015). An opsin suitable for AMD would ultimately need to be tested in the setting of an appropriate model.

## Models of retinal degeneration in AMD

The complexity of AMD is difficult to recapitulate in a single model so several have been developed to represent retinal degeneration (Pennesi et al., 2012). Similarly, there is no single straightforward model of retinal degeneration in GA to evaluate optogenetic therapy (McClements et al., 2020). Therefore, multiple pre-clinical models have been developed to encapsulate different aspects of the spectrum, timeframe, and progression of retinal degeneration.

Several common cell lines have been used to study retinal degeneration in AMD (Forest et al., 2015). Such as induced pluripotent stem cells (iPSCs) generated from AMD patients and human retinal endothelial cells (HRECs) to look at vascular pathways in AMD, and macrophages and microglia cell lines (Malek et al., 2018). The advantages of using such cell lines are to investigate models of RPE dysfunction, study vascular changes, and allow for convenient screening of specific cellular processes such as inflammation or vascular changes. However, cell lines provide a simplified model, have a limited ability to represent genetic diversity given that AMD is a polygenic disease, and lack the following: the complex microenvironment, the interconnections between cell types and three-dimensional tissue architecture of the retina.

Human derived retinal organoids are a useful tool for pre-clinical and *in vitro* testing of optogenetic therapies, offering a complementary approach of functional assessment (Zhong et al., 2014; Quinn et al., 2018). Patient-specific induced pluripotent stem cells (iPSCs) into human retinal organoids allow for modeling AMD.(Xue et al., 2022) For example, *TNF* and *HBEGF*, predicted AMD risk factors, when applied to human organoids resulted in outer retinal pathology with photoreceptor loss (Völkner et al., 2022). This organoid model could reproduce parts of retinal remodeling which is relevant for advanced AMD (Völkner et al., 2022). Another organoid model of AMD was generated (Newcells Biotech) with iPSC-derived RPE from individuals carrying the high-risk *CFH* (Y402H) complement factor variant; it showed microvilli length was decreased, mitochondrial area was increased, number of mitochondria decreased and vacuole number increased with high risk, together suggesting inflammation. It has been reported that there are cellular, structural and functional changes associated with inflammation, stress, and lipid droplet accumulation which resemble AMD. Being of human origin, retinal organoids provide a platform for studying the optimal time for optogenetic intervention. However, retinal organoids remain a simplified model of the complex layers and connectivity of the retina, and do not replicate the *in vivo* or human microenvironment. Also, there can be variability between batches, differentiation and limited longevity (Singh and Nasonkin, 2020).

Mouse models have been engineered to resemble particular aspects of GA and the models are described here with an overview of their feasibility. To study the role of complement system dysregulation, the complement factor H (CFH) knockout mouse model was created which has a deletion in the mouse *CFH* gene (Hoh Kam et al., 2016). The *CFH* gene has been associated with a high risk of GA secondary to AMD and *in vivo* phenotype associated with increased subretinal autofluorescence in *cfh*(−/−) mice (Postel et al., 2006; Coffey et al., 2007). The role of the immune system has been investigated with a *ccl2* and *cx3cr1−/−* mouse model (Tuo et al., 2007). Lipid and cholesterol metabolism abnormalities in an *ApoE*−/− mouse model led to lipid deposition, inflammation and RPE damage which may resemble AMD (Hu et al., 2021). Oxidative stress has also been associated with AMD pathogenesis and older *Sod1−/−* mice have been described to have drusen, thickened Bruch’s membrane and choroidal neovascularization (Imamura et al., 2006). Meanwhile, Nrf2−/− mice developed drusen-like deposits, choroidal neovascularization and sub-RPE deposits of inflammatory proteins (Zhao et al., 2011). The *rpe65* knockout mouse model led to retinal degeneration and visual cycle dysfunction which has been associated with AMD. The *rd1* mouse model (*pde6b* gene mutation) has been well-characterized and widely used for retinal degeneration; it is used most commonly in retinitis pigmentosa since it leads to degeneration of rod photoreceptors (Farber et al., 1994). There is a rapid degeneration of rod photoreceptors with complete loss by post-natal day 18. By contrast the *rd10* mouse model (*pde6b* missense mutation) has a slower progression and less severe phenotype compared to *rd1* (Gargini et al., 2007). A slower progression may provide an opportunistic window for optogenetic therapy to have an effect on structural and functional changes although the milder phenotype may limit the potential efficacy.

Rodent eyes are smaller than humans which means that there is a proportionally larger surface area for viral vector transduction, which could overestimate the efficacy. The smaller size of the rodent eye also limits the volume that can be delivered and dose of vector containing optogenetic therapy. Further, there is a larger lens in rodent eyes which increases the risk of a traumatic cataract, retinal detachment and vitreous hemorrhage which could have a negative impact on the efficacy and safety outcomes (McClements et al., 2020). In mouse models, photoreception is rod predominant with few cones and no fovea which can make cone-based optogenetic therapy more challenging to study, particularly to test in AMD, as this affects the macula, which in humans has a higher proportion of cones. Rodents lack high-acuity vision which limits behavioral studies that estimate visual acuity levels in humans post optogenetic therapy.

To address several of these limitations, larger animal models, such as canine and non-human primates, have been used since the eyes are a similar size to humans (Beltran, 2009). Larger animals have a thicker inner limiting membrane which acts a barrier between the vitreous and neural retina and has a more similar permeability to humans (Yin et al., 2011). Canines have a “area centralis” where there is a high density of cone photoreceptors. It is important in GA when assessing optogenetic therapy to have a model containing a macula because there are different patterns of cellular transduction and functional assessments. Despite having a cone rich area and that the retinal degeneration is slow, the canine model is less practical and more costly compared to rodents.

## Route of delivery

**Figure 5:**
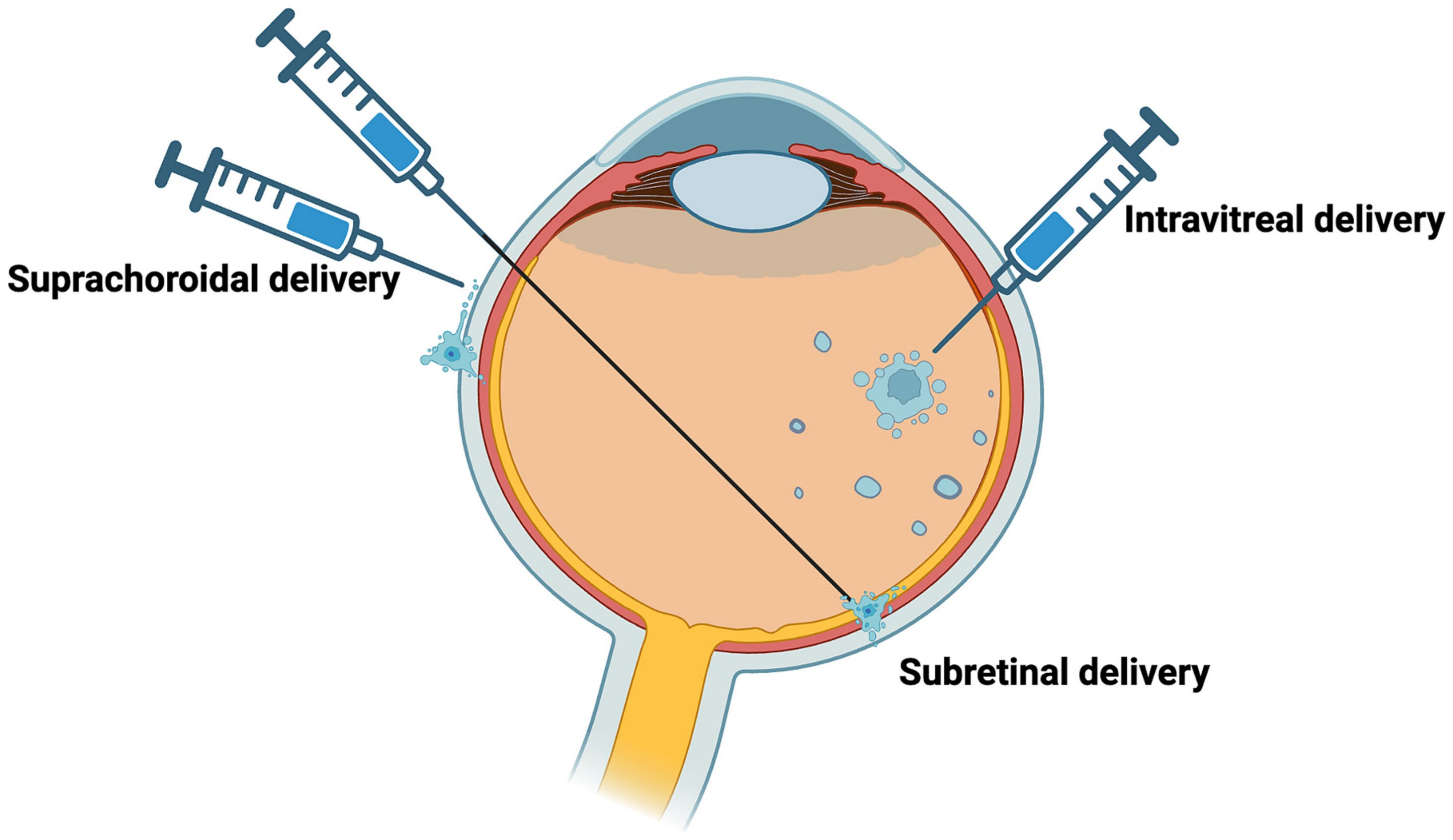
Delivery of potential routes for optogenetic therapy. Potential routes of vector administration include intravitreal, subretinal, suprachoroidal.

Optogenetic treatment can be delivered via gene therapy using several routes of administration (Figure 5). The three main routes of administration include: intravitreal, subretinal, and suprachoroidal injections. Each will be discussed in the context of consideration of optogenetic therapy in AMD.

Intravitreal injection delivers the vector to the vitreous cavity, and this delivery technique is routine practice for anti-vascular endothelial growth factor (anti-VEGF) for the treatment of neovascular AMD. It is a simple procedure and has lower surgical risks compared to subretinal and suprachoroidal injections. In a context of a GA lesion where the outer retina has degenerated, optogenetic therapy targeting inner retinal cells may be more accessible through an intravitreal injection, which is presumably why current optogenetic clinical trials were designed to use this approach. However, there is limited transduction in the inner retinal cells with intravitreal injections because of anatomical barriers such as the ILM and vitreous dilution of AAV (Cehajic-Kapetanovic et al., 2018).

With optogenetic therapy for GA there might be potential adverse effects on the retina outside of the atrophy area, so the administration route of the therapy should be considered carefully to aim at the GA areas specifically, for example by subretinal approach. Improvements of visual function in broader area, outside of the macula, would however be appreciated in many cases of retinitis pigmentosa where photoreceptor degeneration is pan-retinal. This can be achieved by larger and multiple subretinal blebs. Subretinal injection delivers the vector to a virtual, subretinal space between the RPE and neurosensory retina and there is capacity for a greater volume to be administered with each injection. It is a technically more challenging procedure, where following a vitrectomy to gain access to the space while preventing damage from elevated intraocular pressure, a transient bleb is created which can be damaging to the retina. Retinal remodeling with degeneration activates Muller cells that form a scar which can isolate the retina from the subretinal space. Scarring and permeability of the retina may limit the administration of optogenetic therapy in GA to the inner retinal cells. However, in *rd1* and *rd12* mouse models, restoration of visual function has been demonstrated using the subretinal injection approach (De Silva et al., 2017; Suh et al., 2021).

Alternatively, suprachoroidal delivery is targeted to a space formed between the sclera and choroid (Olsen et al., 2006; Patel et al., 2011; Kim et al., 2014). This could allow for the delivery of a vector to a larger surface area compared to the subretinal bleb. In GA, suprachoroidal delivery has been used to explore the use of mesenchymal stem cells which showed choroidal thickening over one year follow up (Habot-Wilner et al., 2019). The potential advantages with suprachoroidal delivery in humans are that it is minimally invasive, minimizes the risk of adverse events such as intraocular pressure elevation and does not need pars plana vitrectomy or iatrogenic retinal detachment to place the vector solution under the retina. Although, there could be limited diffusion as a result of rapid choriocapillaris clearance, possible systemic absorption, lack of immune privilege since it is outside the blood-retinal barrier and the risk of hemorrhage with suprachoroidal delivery (Wu et al., 2023).

## Optogenetic therapy in the clinic trials of retinal degeneration

Optogenetic therapies are already under evaluation in clinical trials for advanced retinal degeneration with gene agnostic approaches (McClements et al., 2020). Current clinical trials are outlined in Table 2. Each clinical trial has either recruited or is currently recruiting patients with advanced retinal degeneration with limited functional vision. The PIONEER study led by Gensight Biologics used AAV2 ChrimsonR-tdT protein (GS030-DP) delivered by intravitreal injection with the use of light-stimulating goggles (GS030-MD). It has been demonstrated that two patients had preliminary partial functional recovery as measured by being able to locate and touch small objects on a table and a good safety profile after 2.5 years follow up (Sahel et al., 2022). The clinical trial led by Allergan also used microbial opsins which have showed some potential in early clinical trials although currently limited by sensitivity, requiring the need for amplification devices. The selection of ChrimsonR instead of ChR2 has been proposed to be safer and more sensitive compared to ChR2. Meanwhile, MCO-010 in Phase I/II clinical trial has demonstrated in vision-guided mobility, shape discrimination and visual acuity. Recently, the 12-month results the phase IIb/III clinical trial were shared and a mean improvement visual acuity improved at 12 months with no serious adverse events (ARVO 2024 abstract #2137).

**Table 2:** Clinical trials involving optogenetic therapy to treat retinal degeneration.

Company	Phase	Drug or device	Opsin	Vector	Status	NCT	Target cell	Vision function	Route	Disease
Allergan	IIa	RST-001	ChR2	rAAV2	Active	*02556736*	Ganglion cells	Worse than HM or *CF* to 20/200	IVI	RP
GenSight Biologics	I/IIa	GS030-DP/MD	ChrimsonR	rAAV2.7 m8	Recruiting	*03326336*	Ganglion cells	Worse than *CF*	IVI	RP
Bionic Sight LLC	I/IIa	BS01	ChronosFP	rAAV2	Recruiting	*04278131*	Ganglion cells	LP in at least one eye	IVI	RP
Nanoscope Therapeutics	I/IIa	vMCO-010	MCO1	rAAV2	Recruiting	*04919473* *05417126*	Bipolar cells	LP or NLP in the study eye	IVI	RP and STGD

RP, retinitis pigmentosa; STGD, stargardt’s disease.

## Conclusion

Irrespective of the structural and functional remodeling in GA secondary to AMD, the inner retina remains intact and may be receptive to optogenetic opsin expression and light-evoked potentials. The surviving cells provide a route to bypass the degenerated photoreceptors to restore vision. There is a need to engineer and optimize an opsin which resembles the function otherwise lost by photoreceptors in GA in AMD. This opsin would need to be highly sensitive, express well in target cells, and restore macular function. It would need to be a human opsin to minimize the immunogenicity. As AAVs have been demonstrated to be safe and effective for retinal gene therapy, they would be the vector of choice for delivery of the opsin. Ganglion cells appear to be a suitable target in GA in AMD since they are better preserved, have improved restored visual response based on amplitude and response latency *in vivo*, and provide a direct pathway to the central nervous system. However, further evidence and a direct comparison is needed between ganglion cells and bipolar cells in the context of GA in AMD to determine the most suitable target cell candidate (Table 2).

While there are many models demonstrating different aspects of AMD pathophysiology, it would be informative to carry out preliminary studies in a retinal organoid model because it can be derived from patient specific iPSC-cells before progressing to an *in vivo* model for further evaluation. The Argus II prosthesis targets ganglion cells and PRIMA prosthesis targets bipolar cells both of which have provided some helpful insights and direct clinical comparison for GA patients based on target cell planning for optogenetic therapy. It was demonstrated that Argus II prosthesis elicited central visual function over GA area in all patients. Meanwhile, there was a 0.9 LogMAR improvement with the PRIMA prosthesis in GA patients (Pixium Vision, 2021). Optogenetics may lead to better functional outcomes and longevity of restored sight compared to retinal implants, since the latter is limited by electrode density, and optogenetics targets residual cells and failure of the microelectrode array due to the inhospitable environment (Maciejewski et al., 2009). A computational model predicted an estimated vision of 20/72 with optogenetics (Ferrari et al., 2020).

There still remains several challenges for translation that need to be addressed for GA. The feasibility of optogenetic therapy in GA compared to retinitis pigmentosa (RP), which has been in clinical trial, was compared (Table 3). The level of visual acuity in selecting patients who could be eligible, consideration of effect of eccentric fixation, and the use of gene therapy vectors with the role that inflammation has in the progression of AMD would need to be explored. There are several optogenetic therapy clinical trials either active or recruiting currently for advanced retinal degeneration, using an intravitreal route of delivery. The safety and efficacy of these trials are awaited in great anticipation.

**Table 3:** Comparison of optogenetics feasibility in late-stage age-related macular degeneration compared to retinitis pigmentosa.

	Geographic atrophy secondary to AMD	Inherited retinal diseases including retinitis pigmentosa
Features	Commonest cause of visual disability in older adults in the high-income countries Affects macula causing irreversible loss of central vision. Aging leads to progressive thickening of the Bruch’s membrane, thinning of the choriocapillaris and hypoxia impairing exchange of macromolecules and debris removal. Inflammation, risk alleles in the complement system, epigenetic, and environmental factors lead to AMD, majority a non-neovascular form. Clinical phenotype shows loss of choriocapillaris, retinal pigment epithelium (RPE), and overlying photoreceptors, termed geographic atrophy if the affected area exceeds 250 μm Inner retina remains relatively preserved	Commonest cause of visual disability in working age adults Affect retinal periphery and/or macula causing irreversible loss of peripheral and/or central vision Heterogeneous group of disorders in terms of genotype, phenotype, age of onset, severity and progression, ultimately leading to loss of photoreceptors Inner retina remains relatively preserved
Advantages of optogenetic therapy	Promising therapy to reverse sight loss Gene agnostic approach which is important since a polygenic disease Current treatment options are limited and none restore visual function	Promising therapy to reverse sight loss Gene agnostic approach which is important since genetically heterogeneous Current treatment options in development are mostly aimed at earlier stages of disease Several optogenetic clinical trials are underway Level of visual function can reach no perception of light at the end stage, so any restoration of visual function is expected to be due to treatment effect Option to restore central and peripheral vision Studies on RP patients demonstrated post-thalamic pathways remain intact which is important for rehabilitation
Disadvantages of optogenetic therapy	Optogenetic therapy has never been trialed in patients with GA Subretinal scarring at the macula may be more severe compared to RP RNFL may be affected in cases complicated by subretinal hemorrhage Visual acuity is not as poor as RP at the end stage and almost never reaches no light perception stage Patients with developed extrafoveal fixation may not be suitable There is a role of inflammation in disease progression and therapy is unlikely to prevent retinal remodeling Generic risks of AAV gene therapy approach include risks of surgery and AAV associated inflammation	May not be possible to restore Clinically significant levels of vision in very end stage cases Role of inflammation and level of retinal remodeling are variable so can lead to unpredictable outcomes Current approaches of vision restoration are aimed at macula and will not restore peripheral vision Generic risks of AAV gene therapy approach include risks of surgery and AAV associated inflammation

The increasing burden of disease of GA highlights the demand for rapid therapeutic advances to restore vision. While slowing the progression is important, vision restoration would improve quality of life and genetic engineering technology with optogenetic therapy shows a great promise as a potential future therapy for GA.

## Author contributions

GAB: Writing – original draft, Writing – review & editing. HS: Writing – review & editing. BN: Writing – review & editing. KX: Writing – review & editing. SRD: Writing – review & editing. SMD: Writing – review & editing. REM: Writing – review & editing. JC-K: Conceptualization, Writing – review & editing.
